# CD47-SIRPα Controls ADCC Killing of Primary T Cells by PMN Through a Combination of Trogocytosis and NADPH Oxidase Activation

**DOI:** 10.3389/fimmu.2022.899068

**Published:** 2022-06-20

**Authors:** Françoise Gondois-Rey, Thomas Miller, Vladimir Laletin, Xavier Morelli, Yves Collette, Jacques Nunès, Daniel Olive

**Affiliations:** ^1^Immunity and Cancer Team, Centre de Recherche en Cancérologie de Marseille (CRCM), Inserm U1068, CNRS UMR7258, Institut Paoli-Calmettes, Aix Marseille University UM105, Marseille, France; ^2^Integrated Chemical and Structural Biology Team, Centre de Recherche en Cancérologie de Marseille (CRCM), Inserm U1068, CNRS UMR7258, Institut Paoli-Calmettes, Aix Marseille University UM105, Marseille, France

**Keywords:** PMN (polymorphonuclear leucocyte), SIRPα, NADPH oxidase, CD47 antibody, ADCC (antibody-dependent cellular cytotoxicity)

## Abstract

Immunotherapies targeting the “don’t eat me” myeloid checkpoint constituted by CD47 SIRPα interaction have promising clinical potential but are limited by toxicities associated with the destruction of non-tumor cells. These dose-limiting toxicities demonstrate the need to highlight the mechanisms of anti–CD47-SIRPα therapy effects on non-tumor CD47-bearing cells. Given the increased incidence of lymphopenia in patients receiving anti-CD47 antibodies and the strong ADCC (antibody-dependent cellular cytotoxicity) effector function of polymorphonuclear cells (*PMNs*), we investigated the behavior of primary PMNs cocultured with primary T cells in the presence of anti-CD47 mAbs. PMNs killed T cells in a CD47-mAb–dependent manner and at a remarkably potent PMN to T cell ratio of 1:1. The observed cytotoxicity was produced by a novel combination of both trogocytosis and a strong respiratory burst induced by classical ADCC and CD47-SIRPα checkpoint blockade. The complex effect of the CD47 blocking mAb could be recapitulated by combining its individual mechanistic elements: ADCC, SIRPα blockade, and ROS induction. Although previous studies had concluded that disruption of SIRPα signaling in PMNs was limited to trogocytosis-specific cytotoxicity, our results suggest that SIRPα also tightly controls activation of NADPH oxidase, a function demonstrated during differentiation of immature PMNs but not so far in mature PMNs. Together, our results highlight the need to integrate PMNs in the development of molecules targeting the CD47-SIRPα immune checkpoint and to design agents able to enhance myeloid cell function while limiting adverse effects on healthy cells able to participate in the anti-tumor immune response.

**Graphical Abstract:**
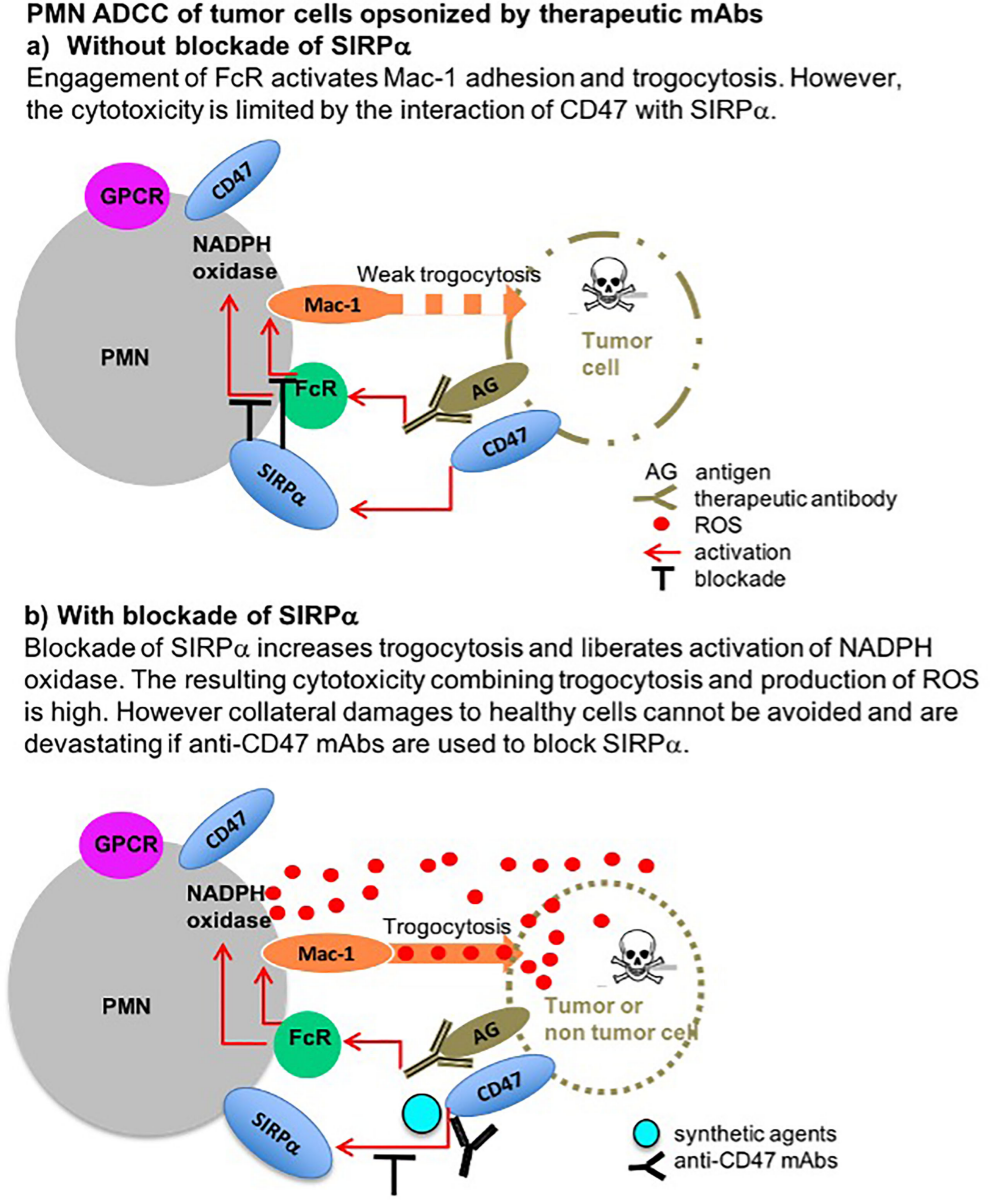


## Highlights

We show that potent cytotoxicity by PMN occurs through a novel combination of trogocytosis and ROS regulated by CD47-SIRPα. We also show that anti-CD47 antibody therapy risks collateral damage by opsonizing and directing this cytotoxicity to healthy T cells.

## Introduction

The ability to manipulate patients’ immunity with antibodies has changed the therapeutic outlook for cancer. Strategies based on the induction of antibody-dependent cell cytotoxicity (ADCC) with antibodies targeting tumor antigens like Rituximab in B-lymphomas and Trastuzumab in breast cancer have demonstrated clinical efficacy. More recently, strategies based on the blockade of the T cells inhibitory checkpoints CTLA-4 and PD-1/PD-L1 with mAbs showed spectacular efficacy but were limited to certain tumor types with high mutational burdens and T cell infiltration and associated side effects (1). To create therapies with broader efficacy, strategies targeting the myeloid checkpoint and signal regulatory protein α (SIRPα) were developed to enhance myeloid ADCC induced by therapeutic antibodies (2, 3).

SIRPα controls phagocytosis when engaged by its ligand CD47, a molecule widely expressed in most cell types (4). This interaction constitutes the “don’t eat me” regulatory axis. Inhibition results in part from the immunoreceptor tyrosine–based inhibitory motif (ITIM) phosphorylation of SIRPα cytoplasmic tail that prevent modifications of the membrane, leading to the formation of the phagocytic cup (5). The regulation of SIRPα signaling in PMN is known to be sensitive to their activation where IL-17 stimulation results in cleavage of the ITIM signaling domain of SIRPα (6). SIRPα also inhibits the activation of Mac-1, the integrin required for the spread and adhesion of myeloid cells on their target (7, 8). Mac-1 is a heterodimer of CD11b (αM) and CD18 (β2) integrins but only CD18 is required in ADCC-mediated adhesion (9). Whereas macrophages are capable of whole-cell phagocytosis, polymorphonuclear cells (PMNs) ingest parts of the target cell in a mechanism called trogocytosis (10, 11). In ADCC, trogocytosis is sufficient to induce necrotic cell death resulting from lytic processes but requires high ratios of PMNs per target cell (10, 12).

PMNs are mainly known for their capacity to degranulate toxic molecules accumulated during their maturation or produce toxic reactive oxygen species (*ROS*) upon the “respiratory burst” within minutes of stimulation to fulfill their killing mission (13). The nicotinamide adenine dinucleotide phosphate (NADPH) oxidase is a multi-subunit enzyme complex that, once assembled at the PMN membrane, generates superoxide as a precursor for other ROS (H_2_O_2_, HOCl), released in the milieu as an antimicrobial agent. However, ROS are also toxic to host cells and this process must be tightly controlled. NADPH oxidase is activated through membrane G protein–coupled receptors (*GPCR*) that sense the various molecules of the milieu, such as bacterial compounds (fMLP and LPS) (14), but little is known about its control. Recently, SIRPα was shown to be involved in the control of NADPH oxidase by inhibiting the expression of the gp91^Phox^ subunit, a membrane component of NADPH oxidase complex, in immature cells (15). This inhibition required engagement of SIRPα by CD47 and signaling by the cytoplasmic tail of SIRPα. Blockade of SIRPα engagement resulted in enhanced production of ROS. However, NADPH oxidase and ROS are not known to be involved in ADCC (10).

CD47 is a widely expressed signaling receptor and marker of “self” involved in many biological processes through its interaction with its ligand thrombospondin-1 (*TSP-1*), an inflammatory protein that promotes migration and activation of cells (16). Different epitopes of CD47 are involved in the interaction of CD47 with TSP-1 and SIRPα (17). CD47 interacts also with SIRPγ, a molecule only expressed in T cells (18). CD47 is known to signal through its lateral association with integrins and GPCR (19). For example, triggering of CD47 induces endothelial cell spreading on RGD sequences through the lateral association of CD47 with β3 integrins and adhesion of T cells on LDV sequences through the lateral association with β1 integrins (20). An interaction of CD47 with Mac-1 was recently described as one of the mechanisms involved in the fusion of macrophages (21).

The overexpression of CD47 on tumor cells suggested that blockade of the “don’t eat me” checkpoint could synergize with therapeutic mAbs (16) to enhance the elimination of tumors by myeloid cells (22). Antibodies blocking the CD47-SIRPα interaction increased phagocytosis of macrophages (22, 23) and cytotoxicity of PMNs (12), inhibited tumor engraftment (22), and eliminated pre-existing tumors in mice (24). Although both can block the “don’t eat me” interaction, anti-CD47 mAbs were more efficient than anti-SIRPα mAbs (24). This higher efficiency was thought to result from the additional ADCC resulting from the opsonization of the target with anti-CD47 mAbs (4, 12).

Despite their promising pre-clinical results, the clinical progress of anti-CD47 mAb therapies have been limited by on-target, non-tumor toxicities including anemia, neutropenia, thrombocytopenia, and lymphopenia (25). We had previously identified the CD47-SIRPα immune checkpoint and SIRPα activity as key determinants of low-density PMN-MDSCs (myeloid-derived suppressive cells) cytotoxicity toward healthy T cells (26). Low-density PMN-MDSCs share immune-suppressive capacities while they are composed of heterogeneous populations of immature and mature cells having acquired low-density properties after activation (27). We investigated the effect of the SIRPα-blocking anti-CD47 mAb (clone CC2C6) on high-density mature PMN-mediated T cell cytotoxicity. By investigating their ADCC on primary T cells, we found that blockade of SIRPα engagement on PMNs resulted in an important cytotoxicity sustained not only by an enhancement of trogocytosis but also by induction of a strong respiratory burst, resulting in suppression of T cells.

## Materials and Methods

### Cells

Blood samples were obtained from healthy donors (EFS, Etablissement Français du Sang, Marseille, France). High-density PMNs were separated from peripheral blood mononuclear cells (PBMC) by centrifugation on Ficoll-Hypaque gradients. Red cells were eliminated with RBC lysing buffer (eBioscience, ThermoFischer, France). PMNs were kept at 4°C in PBS supplemented with Ca^2+^ 1 mM and Mg^2+^ 1 mM. The purity of the PMN preparations was routinely between 70%–90%, contaminants were T cells, and monocytes were absent (**Figure S1A**). T cells were separated from frozen or fresh PBMC using CD3^+^ magnetic beads (Miltenyi Biotech, Germany), and the purity of preparations was above 95% (not shown). A weak death of T cells (10%–20%) was observed after overnight culture (not shown). Raji B cell lines were obtained from ATCC. For the Jurkat T cell line, JA16 was initially subcloned in the lab (28), and JINB8 is a CD47deficient Jurkat cell line (29). Cells were cultivated in RPMI 1640 medium supplemented with 10% foetal calf serum and antibiotics.

### Antibodies and Peptides

Purified anti-CD47 mAbs clones CC2C6 and 2D3 (BioLegend) and clone B6H12 (BD Biosciences) and purified anti-SIRPα mAbs (clone SE5A5) and G1 isotype mAbs were used at 10 μg/ml (BioLegend). Anti–Mac-1 was reconstituted by mixing anti-CD11b (clone ICRF44) and CD18 (clone TS1/18) mAbs at a final concentration of 10 μg/ml for each (BioLegend). Anti-CD3 mAbs (clone UCHT1) were prepared in the laboratory and used at 10 μg/ml. Recombinant SIRPα was prepared as in (30) and was used as a monomer or multimerized with Neutravidin at a saturating concentration of 5 μM. RGDS and LGDP were used, respectively, at 40 μg/ml and 100 μg/ml (Sigma Aldrich Merck). 4N1K, a CD47-binding domain adhesive peptide derived from TPS1, was used at 10 μM (Eurogentec). CC2C6-F(ab)’2 was prepared using F(ab’)2 Preparation Kit (Pierce) as described by the manufacturer using an optimized 1-h digestion time at 37°C. Digestion was verified by sodium-dodecyl-sulfate polyacrylamide gel electrophoresis (SDS-PAGE), and CD47 binding activity was measured against the parent CC2C6 using AlphaScreen as described in (30).

### Cytotoxicity Assay

Targets were stained with CellTrace™ Violet (Life Technologies, France). Counting beads (BD Biosciences, France) were added to target cell suspensions. Cocultures were set using PMNs and T cells from different donors at ratios ranging from 1/1 to 5/1 PMNs to target and incubated overnight at 37°C in culture medium. An aliquot of the coculture was stained and analyzed by flow cytometry immediately after mixing to verify ratios and after overnight incubation to evaluate cytotoxicity by counting live targets. Target counts were standardized to counting beads and either compared to counts of control targets cultured overnight without PMNs or to control coculture with PMNs. T cells did not proliferate during overnight culture, Raji cells slightly proliferated, and Jurkat and JINB8 proliferated by a factor of 3–5. PMNs remained alive during overnight culture but showed a trend to display an activated phenotype characterized by a decrease of SSC and an increase of FSC and depending on the stimulation counts importantly decreased (shown in **Figure S2B**). This uncontrolled weak activation of PMNs during their preparation was previously described (31) and resulted in spontaneous cytotoxicity or protection of T cells from spontaneous death ranging between 50% and 150%. Experiments were excluded where this effect was over 20% to focus on the effects of antibodies. Inhibition of respiratory burst was performed by adding 50 μg/ml of catalase to digest H_2_O_2_ and 10 μM of Diphenyleneiodonium chloride (DPI) to inhibit NADPH oxidase (Sigma-Aldrich, Merck) during the overnight coculture. DPI could not be used with cell lines as a target because it inhibited cell proliferation. Because cell lines proliferated during the overnight incubation cytotoxicity could no more be quantified as it was based on the absolute counting of cells.

### Flow Cytometry

**Figure 1:**
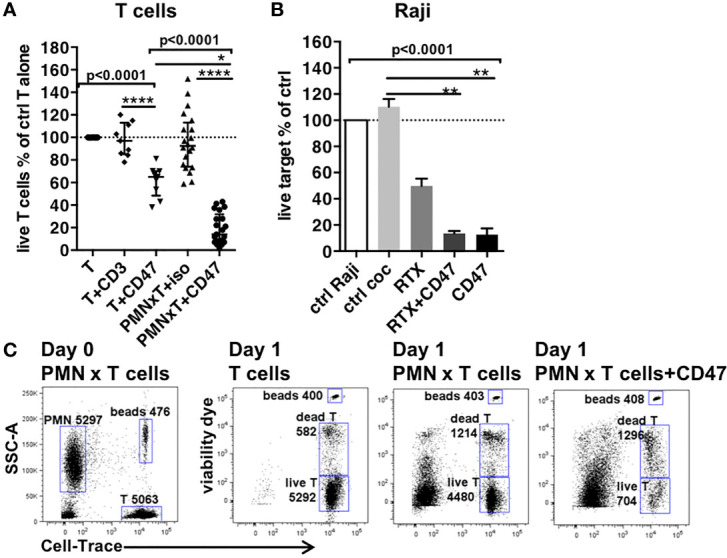
Anti-CD47 mAbs induce killing of primary T cells by PMNs. **(A)** Cytotoxicity to primary T cells induced by anti-CD47 mAb clone CC2C6 alone or in the presence of PMNs (n = 9–25 different donors; ratio of PMN to T cells = 2). Iso, isotype; CD3, anti-CD3 mAbs; CD47, anti-CD47 mAbs. **(B)** Induction of PMNs’ cytotoxicity to Raji lymphoma B cells by combinations of Rituximab (RTX) *plus* anti-CD47 mAb clone CC2C6 (CD47) (n = 3; ratio of PMN to target = 3). For **(A, B)**, cytotoxicity is represented by the % of live targets in indicated conditions compared to targets cultivated overnight alone. Median and IQR (interquartile range) are shown. *P*-values from Kruskall–Wallis test is indicated on top of groups, *P*-values from Dunn’s multiple comparison post-test on top of pairs: *P < 0.05; **P < 0.01; ****P < 0.0001. **(C)** Cytotoxicity assay. The left plot shows the coculture of PMN with Cell-Trace stained T cells on day 0. The following plots show cell-trace and viability staining of indicated cocultures after overnight incubation. Gates and counts of beads, live and dead Cell-Trace–stained T cells are shown.

Cell suspensions were stained with Near-IR LIVE/DEAD™ (Life Technologies), CD3-PC5 or CD19-PC5, and CD11b-PE when indicated (BD Biosciences). Samples were acquired on a FORTESSA cytometer (BD Biosciences). Data were exported and analyzed with FlowJo (RRID : SCR_008520; version 9-2, MacOS X). Counting beads and cells were gated on forward scatter-area/side-scatter area- (FSC-A/SSC-A) (shown in **Figure 1C**). Doublets were excluded on FSC-A/FSC-H. Dead cells were excluded on the expression of the viability dye (shown in **Figure S1A**). PMNs were gated as SSC^hi^ cells and CD11b expression. For analysis of trogocytosis of PMNs, target T cells were excluded on the expression of CD3. For cytotoxicity assays, targets (T cells, Jurkat T cell-lines, and Raji B lymphoma cell-line) were gated on SSC^low^FSC^hi^, cell-trace, and CD3 or CD19 expression.

### Trogocytosis

Target cells were stained with the membrane-dye PKH67 (Sigma-Aldrich) and cocultured with PMNs at ratios ranging from 1:1 to 3:1 for 3 h. Trogocytosis was also analyzed in PMNs recovered from cytotoxicity assays after an overnight incubation with targets stained with CellTrace. Percentages of trogocytosis were determined by the expression of the T cell dye in PMNs after setting gates on PMNs cultured alone.

### Intracellular Production of ROS

PMNs were stained with 25 μM Dihydrorhodamine 123 (DHR, Sigma-Aldrich) and cultured for 1–1.5 h in the presence of catalase at 50 μg/ml (32). Lipopolysaccharide (LPS, 100 ng/ml) *plus* N-Formylmethionyl-leucyl-phenylalanine (fMLP, 5 μM) and monoclonal antibodies at 10 μg/ml were added immediately after DHR staining. Samples were acquired on a FORTESSA cytometer, DHR was analyzed in the 525/50 channel and the percentage was determined by setting the gate on un-stimulated stained cells.

### Statistics

Statistical graphics were performed with Prism 6 (RRID : SCR_005375) software. Mann–Whitney, Wilcoxon matched pair non-parametric test or Kruskall–Wallis test followed by multiple comparison Dunn’s post-test to compare variables between groups were used as indicated.

### Data Availability

The data generated in this study are available within the article and its supplementary data files.

## Results

### Anti-CD47 mAbs Induce Killing of Primary T Cells by PMNs

To investigate the potential for CD47-dependent PMN killing of primary T cells, we treated cocultures with anti-CD47 mAb CC2C6 at a PMN to T cell ratio of 2:1. Overnight coculture with PMNs alone resulted in a variability of 50%–150% of T cells survival. The introduction of CC2C6 to the PMNs T cell coculture resulted in a much stronger cytotoxicity (mean survival of 19% of control-treated T cells; **Figures 1A**, **S2B**). We noted that CC2C6 had a direct weak cytotoxicity to T cells as previously shown with other antibodies to CD47 (33). As expected, percentages of dead T cells in coculture with PMNs and anti-CD47 mAbs were increased as compared to control T cells cultivated alone or with PMNs without anti-CD47 mAbs (**Figure 1D**) (67% vs. 15% and 22%, respectively). However, the most striking difference was the significant decrease of total cells suggesting that cytotoxicity manifested as necrosis rather than apoptosis, as reported for PMNs induced killing (10) but the possibility that dead T cells were removed by PMNs’ efferocytosis cannot be excluded as well.

The potent CD47 mAb-induced PMN cytotoxicity was not limited to primary T cells as we observed similar cytotoxicity with Raji tumor cells (Raji) as targets. At a ratio of PMN to a target of 3:1, RTX induced the killing of 50% of the Raji tumor cells (Raji) (**Figures 1B**, **S2A**). The addition of anti-CD47 mAb to RTX reduced the survival of Raji to 14% of control, but the anti-CD47 mAb alone reduced cell survival to 13%. The similarity of cytotoxicity induced by the anti-CD47 mAb whether alone or with RTX suggested that they were mainly responsible for the target cell death when used in the combination. Because of the uniquely potent nature of the cytotoxicity observed for PMN in the presence of an anti-CD47 antibody, we sought to investigate its mechanism.

### Trogocytosis *plus* CD47-SIRPα Blockade Are Insufficient to Explain the Enhanced Cytotoxicity of PMNs in Anti-CD47 mAb-Triggered ADCC

Anti-CD47 mAbs are capable of exerting cytotoxic functions *via* a variety of mechanisms. They can simultaneously opsonize T cells to activate PMNs’ FcR providing “eat me” signals while also inhibiting “don’t eat me” signals resulting from the engagement of PMNs’ SIRPα by CD47 on T cells. PMN-mediated toxicity *via* ADCC was recently shown to proceed almost exclusively by trogocytosis of the target cell membrane and to be regulated by SIRPα (10). We reconstituted these mechanisms with anti-CD3 mAbs to opsonize T cells and anti-SIRPα mAbs to block “don’t eat” signals while comparing trogocytosis by PMNs and cytotoxicity to T cells.

**Figure 2:**
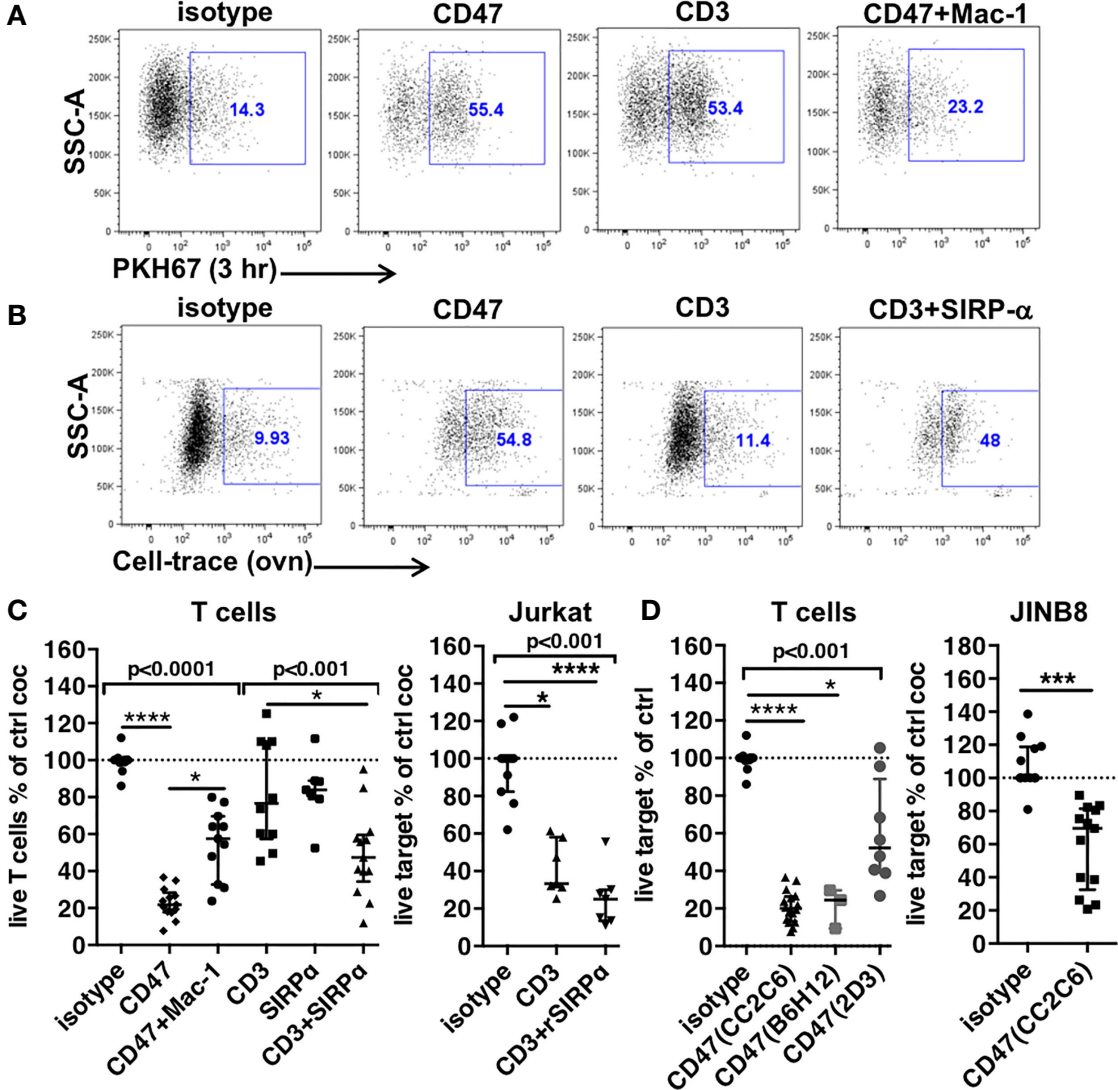
Trogocytosis and cytotoxicity in PMNs’ ADCC. **(A)** Expression of the PKH67 T cells membrane dye in PMNs after a 3-h coculture in the presence of indicated mAbs. **(B)** Uptake of Cell-Trace-stained T cells components in PMNs after overnight coculture in the presence of mAbs. For **(A, B)**, PMNs are gated after exclusion of doublets and dead cells. **(C, D)** Cytotoxicity of PMNs to targets indicated on top of graphs, evaluated by the percentages of live targets compared to control after overnight coculture. N = 6–12 different donors, ratios of PMNs to target = 1–3. CD3, anti-CD3 mAbs; CD47, anti-CD47 mAbs clone CC2C6, B6H12, or 2D3 as indicated; Mac-1, anti-CD11b+anti-CD18 mAbs. SIRPα, anti-SIRPα mAbs. rSIRPα, recombinant SIRPα protein. Median and IQR are shown. *P*-values from Kruskall–Wallis test indicated on top of groups, *P*-values from Dunn’s multiple comparison post-test on top of pairs: *P < 0.05; ****P < 0.001. For **(D)**, *P*-values from Wilcoxon matched pair test is shown.

T cells were stained by PKH67 a lipophilic dye that accumulates in the plasma membrane and whose transfer to non-labeled cells indicates trogocytosis (34). Transfer of the membrane dye to PMNs was analyzed by flow cytometry after a 3-h incubation. Similar percentages of PKH67^+^PMNs were found with anti-CD47 and anti-CD3 mAb treatment (59% and 60%, respectively, **Figure 2A**) suggesting an equivalent interaction of PMNs with T cells whether opsonized with anti-CD3 or anti-CD47 mAbs. To control for non-specific cellular adhesion or ADCC mediated adhesion, we included an anti–Mac-1 mAb that would block specific ADCC mediated adhesion *via* integrins α_M_/β_2_. Anti–Mac-1 mAbs inhibited PMNs trogocytosis induced by anti-CD47 mAbs. Given the lateral interactions of CD47 with integrins (17), we verified whether β1 and β3 (as control) integrins were involved, but only antibodies to the β2 integrin CD18 inhibited anti-CD47 mAbs-induced trogocytosis, as reported for PMNs’ ADCC (**Figure S3B**).

These results suggested that this acute trogocytosis was not controlled by CD47-SIRPα signaling but *via* FcR activation. We further investigated trogocytosis in different experimental conditions using CellTrace Violet–labeled T cells incubated overnight with PMNs [as in (10)] (**Figures 2B**, **S3A**). The uptake of CellTrace in PMNs triggered by anti-CD3 mAbs was not different from control (11% vs. 9.9%, respectively). Blockade of CD47-SIRPα interaction with anti-SIRPα targets only the PMNs as T cells do not express SIRPα. Anti-SIRPα antibodies increased trogocytosis to a level similar to anti-CD47 mAbs (48% and 54.8%, respectively), demonstrating CD47-SIRPα regulation of PMNs’ trogocytosis and again showing an equivalent interaction of PMNs with T cells.

Next, we compared the cytotoxicity induced in overnight cocultures. At ratios ranging from 1 to 3:1, anti-CD47 mAbs-ADCC induced by clone CC2C6 or clone B6H12 decreased T cells viability of a factor of 5 (22% survival) (**Figures 2C, D**, **S2B**). Moreover, B6H12 is not directly cytotoxic (35) showing that the equivalent effect of the anti-CD47 antibodies is due to ADCC. Anti–Mac-1 mAbs, while fully inhibiting trogocytosis, only partially restored T cells survival (54% vs. 22%). This suggested that adhesion-mediated ADCC was involved in cytotoxicity but does not account for the complete effect. Consistent with a trogocytosis mechanism, PMNs’ cytotoxicity induced by anti-CD3 mAbs was weak but became significant when anti-SIRPα mAbs were added (82% vs. 50% survival, respectively). Similarly, blockade of SIRPα with a recombinant SIRPα protein (rSIRPα) [as in (36)] increased cytotoxicity of PMNs to Jurkat T cells opsonized with anti-CD3 mAbs by a factor of 1.6-fold (41% vs. 25%, respectively) (**Figure 2C**). In the two systems, however, blockade of SIRPα did not result in the same level of cytotoxicity induced by anti-CD47 mAbs. Although the direct effect of anti-CD47 mAbs on T cells must slightly contribute to the whole effect in the presence of PMNs (see **Figure 1B**), the combination of anti-CD3 *plus* anti-SIRPα mAbs seemed unable to induce similar level of PMN-mediated cytotoxicity despite inducing similar trogocytosis.

Thus, trogocytosis is not sufficient for cytotoxicity. Our results suggested a harmless propensity of PMNs to engulf parts of the membrane of opsonized cells not followed by death of targets. Overnight trogocytosis of internal cell components was better correlated to cytotoxicity but not quantitatively. These results suggested that another mechanism than trogocytosis *plus* blockade of the engagement of SIRPα was involved in the cytotoxicity induced by anti-CD47 mAbs. To demonstrate more directly this hypothesis, we used an anti-CD47 mAb targeting an epitope outside the interaction site with SIRPα (2D3) to induce ADCC against T cells and a CD47-deficient Jurkat T cell line as target for PMNs in the presence of anti-CD47 mAb CC2C6 (**Figures 2D**, **S1B**, and **S2C**). Although a modest effect was expected, PMNs displayed cytotoxicity in the two systems demonstrating the contribution of additional mechanisms likely involving CD47 on PMNs in the strong cytotoxicity.

### Anti-CD47 mAbs Induced Strong ROS Production in PMN

**Figure 3:**
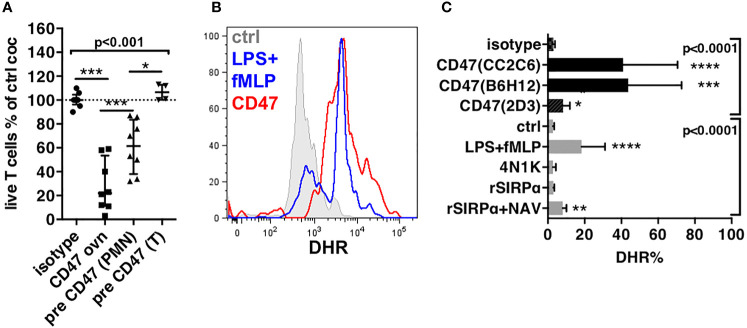
Induction of production of ROS in PMNs. **(A)** Effect of pre-treatment of cells by the anti-CD47 mAb clone CC2C6 on cytotoxicity of PMNs to T cells. Cytotoxicity is represented by the percentages of live T cells compared to control coculture (ctrl coc). Pre-CD47, pre-treatment 30 min at 4°C. CD47 ovn, anti-CD47 mAb during overnight culture, n = 8, median and IQR. **(B)** Histograms of DHR staining in PMNs after exclusion of dead cells and doublets after 1-h stimulation. **(C)** Percentages of expression of DHR in PMNs after 1-h stimulation. N = 5–15 for antibodies, n = 5 for recombinants proteins or peptides. Gates are set on unstimulated stained cells. *P*-values from Kruskall–Wallis test indicated on top of groups, *P*-values from Dunn’s multiple comparison post-test on top of pairs: *P < 0.05; **P < 0.01; ***P < 0.001; ****P < 0.0001.

To address the role of PMN CD47 engagement vs. T cell CD47 engagement in cytotoxicity, we pre-treated PMNs or T cells for 30 min with the anti-CD47 mAb CC2C6 then washed the antibody before coculture with T cells. Only pre-treatment of PMNs resulted in a significant cytotoxicity to T cells, however weaker than that obtained when the antibody was present throughout the coculture (57% and 18%, respectively, **Figure 3A**).

Because PMN are potent producers of ROS we investigated whether PMNs stimulated by anti-CD47 mAbs produced ROS using DHR staining. LPS *plus* fMLP were used as a positive control for ROS induction, and isotype as a negative control. Anti-CD47 mAb CC2C6 induced a rapid increase of DHR fluorescence in PMNs similar to LPS *plus* fMLP (**Figures 3B** and **S4A**). CC2C6 was described to induce hemagglutination through clustering of CD47 (35, 37). To evaluate whether this mechanism accounted for activation of NADPH oxidase, we used B6H12 an anti-CD47 mAb that similarly targeted the epitope responsible for interaction with SIRPα but did not induce clustering of CD47. B6H12 also induced a strong production of ROS, suggesting that activation of NADPH oxidase was rather resulting from triggering of CD47 than its clustering. This hypothesis was consistent with the known lateral association of CD47 with GPCR involved in NADPH oxidase activation (19). Consistently, the 2D3 mAb also induced ROS, however, to a lower level (**Figures 3C** and **S4A**). Likewise, weaker binding recombinant ligands of CD47 like the 4N1K peptide (38) and recombinant SIRPα (30) failed to induce ROS except when rSIRPα was multimerized with neutravidin suggesting nevertheless that avidity or membrane clustering might be important factors of direct ROS induction through CD47 (**Figure 3C**). Alternatively, the weak levels reached rather suggested that other mechanisms than direct engagement of CD47 on PMNs were involved.

### Production of ROS During PMN ADCC is Activated by FcR Stimulation and Tightly Controlled by SIRPα

**Figure 4:**
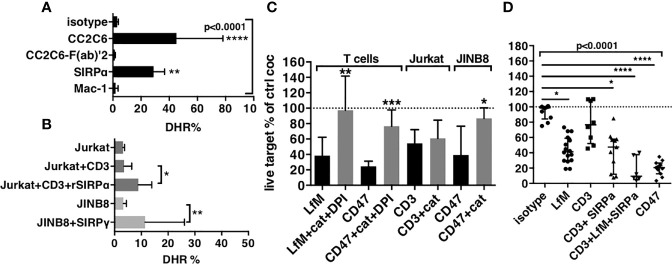
Production of ROS is stimulated in ADCC and contributes to cytotoxicity. **(A)** Stimulation of ROS in PMNs by mAbs to PMNs. **(B)** Stimulation of ROS in PMNs by cocultures with targets and mAbs. For **(A, B)**, median and IQR of percentages of expression of DHR in PMNs after 1-h stimulation. For **(A)**, n = 6–15 different donors. *P*-values from Kruskall–Wallis test indicated on top, *P*-values from Dunn’s multiple comparison post-test on top of pairs: **P < 0.01; ****P < 0.0001. For **(B)**, n = 6–10. *P*-values of Wilcoxon matched pair test: *P < 0.05; **P<0.01. **(C)** Inhibition of cytotoxicity by catalase (cat), in the presence of DPI when indicated, n = 6–25. Median and IQR, *P*-values of Wilcoxon matched pair test: *P < 0.05; **P < 0.01; ***P < 0.001. **(D)** Reconstitution of the cytotoxicity induced by anti-CD47 mAb CC2C6 (CD47) using anti-CD3 (CD3) + anti-SIRPα (SIRPα) + LPS + fMLP (LfM), n = 6–14. Median and IQR, *P*-values from Kruskall–Wallis test indicated on top, *P*-values from Dunn’s multiple comparison post-test on top of pairs: *P < 0.05; **P < 0.01; ****P < 0.0001.

Anti-CD47 mAbs CC2C6, B6H12, and 2D3 have an intact Fc domain; thus, the contribution of FcR in the induction of ROS could not be excluded. This scenario might result from reciprocal FcR activation in PMNs, or activation through trimolecular complex between known as the scorpion effect (39) previously observed with anti-SIRPα antibodies on the macrophage. We investigated the contribution of FcR using an F(ab’)2 fragment of CC2C6 or whole antibodies targeting other molecules on PMNs. CC2C6-F(ab)’2 and anti–Mac-1 mAbs failed to induce ROS, whereas anti-SIRPα mAbs induced significant levels (**Figures 4A** and **S4A**). Thus, FcR stimulation induced ROS in PMNs only in the context of the simultaneous blockade of SIRPα. Consequently, FcR activation of NADPH oxidase was simultaneously and strictly controlled by the engagement of SIRPα, a feature not yet described in PMN.

To demonstrate this hypothesis, we investigated intracellular induction of ROS in short cocultures of PMNs with targets (**Figures 4B** and **S4B**). Coculture with Jurkat opsonized by anti-CD3 mAbs did not increase DHR in PMNs (5% and 4%, respectively), whereas the presence of rSIRPα (to block interaction with SIRPα) significantly increase DHR to 10%. Similarly, coculture of PMNs with CD47-deficient target cells (JINB8) opsonized by anti-SIRPγ mAbs significantly increased DHR in PMNs to 20%. These results suggested that stimulation of PMNs through FcR induced ROS but only when PMN-SIRPα was not engaged by target cell CD47, either by blockade of CD47 with rSIRPα or by the absence of CD47 on target.

Next, we evaluated the contribution of ROS in cytotoxicity induced by PMNs’ ADCC on T cells using catalase, an H_2_O_2_ scavenger *plus* diphenyleneiodonium chloride (DPI), an NADPH oxidase inhibitor, to counteract ROS effects during coculture. LPS *plus* fMLP used to induce ROS by a pathway independent of ADCC resulted in a significant cytotoxicity fully blocked by catalase plus DPI, whereas the cytotoxicity of anti-CD47 mAbs was significantly but partially reduced (56% vs. 26% survival, respectively, **Figure 4C**). Only catalase could be used in assays with Jurkat and JINB8 because DPI is toxic to these cell lines. Catalase alone showed no inhibition of the cytotoxicity induced by anti-CD3 mAbs on Jurkat T cells, suggesting no contribution of ROS but alternatively no inhibition of NADPH oxidase. Conversely, the almost full inhibition of the cytotoxicity of anti-CD47 mAbs on CD47-deficient target cells (JINB8) by catalase showed both the primary use of ROS in this cytotoxicity and that catalase was sufficient for its inhibition (**Figure 4C**).

These results suggested that ROS produced during ADCC in the context of blockade of SIRPα contributed to the cytotoxicity of PMNs. Finally, we verified our hypothesis by using together anti-CD3 mAbs to induce ADCC, anti-SIRPα mAbs to block the inhibitory checkpoint, and LPS *plus* fMLP to induce ROS. This combination reached the levels of cytotoxicity induced by anti-CD47 mAbs, suggesting the important contribution of ROS in the process (**Figures 4D** and **S3B**).

## Discussion

In the context of the toxicities generated by blockade of the SIRPα-CD47 checkpoint in cancer therapies, we sought to address the role of PMN in the lymphopenia observed with anti-CD47 antibody treatment (25). We found that the strong PMN-mediated ADCC induced by the anti-CD47 mAbs CC2C6 was sustained not only by trogocytosis but also by a strong respiratory burst, both controlled by SIRPα. The cooperation of both results in a uniquely efficient killing of T cells in a low effector cell to target cell ratio.

The anti-CD47 mAb clone CC2C6 can induce a weak T cells death through direct triggering (33). In coculture with PMNs, this antibody can stimulate FcR on PMNs and simultaneously blocks CD47 interaction with SIRPα, resulting in potent ADCC (4, 40). In this regard, we found that PMNs killed leukemic B cells in the presence of anti-CD47 antibodies regardless of the presence of the well-known ADCC antibody Rituximab. This result suggested that anti-CD47 mAbs alone were sufficient for a cytotoxic response and explained why PMNs also killed T cells. However, the reconstitution of this scenario using a T cell opsonizing antibody (anti-CD3) that induces ADCC *plus* anti-SIRPα mAbs or recombinant SIRPα protein to block the “don’t eat me” checkpoint failed to induce such strong killing suggesting that another mechanism must contribute to the cytotoxicity induced by anti-CD47 mAbs.

The most likely hypothesis was that this additional mechanism was triggered by the interaction of the antibody with CD47 on PMNs. This was addressed by measuring the anti-CD47–induced cytotoxicity of PMN cells in coculture with CD47-deficient T cells (Jurkat T cell clone JINB8). Although less toxicity was observed when compared to CD47^+^ T cells, we still observed a significant increase in killing as compared to the control treatment. These observations suggest that targeting CD47 on PMN is sufficient to activate cytotoxic mechanisms.

We investigated whether respiratory burst, the most common mechanism used by PMNs to efficiently kill their targets in a short time, was involved. Not surprisingly, ROS contributed to the strong killing of T cells by PMNs induced by anti-CD47 mAbs. This was demonstrated by i) direct induction of ROS in PMNs by stimulation with anti-CD47 mAbs, ii) partial blockade of cytotoxicity by catalase and DPI, and iii) reconstitution of equivalent cytotoxicity by induction of ADCC with anti-CD3 mAbs *plus* blockade of SIRPα with antibodies *plus* stimulation of ROS with LPS and fMLP.

The next question was to determine how anti-CD47 mAbs stimulated NADPH oxidase in PMNs. The known lateral association of CD47 with GPCR, themselves involved in NADPH oxidase activation (14, 19), suggested at first that binding of CD47 by antibodies was involved. This hypothesis was confirmed by the induction of ROS by all anti-CD47 mAbs tested, including 2D3 that targets an epitope outside the interaction site with SIRPα (35, 40). Monomeric ligands failed to induce ROS, but a higher affinity multimer of the recombinant SIRPα protein was obtained with neutravidin [KD = 16 nM (36),], suggesting that affinity or CD47 clustering might be critical. Indeed, CC2C6 reportedly induced cell aggregation through CD47 clustering (37), but B6H12, although not described to possess such property, was efficiently activating NADPH oxidase as well suggesting that clustering of CD47 may not be a determinant. The weak levels reached with SIRPα rather than antibodies showed that activation of ROS through the direct triggering of CD47 was limited and prompted us to consider an alternative mechanism based on stimulation of FcR either by reciprocal interactions between PMNs or by the formation of trimolecular complexes with FcR on each cell (39). This hypothesis was addressed by the use of the F(ab)’2 fragment of CC2C6 to induce ROS. We found that induction of ROS occurred only with antibodies that simultaneously engage FcR and blocked SIRPα (CC2C6, B6H12, and anti-SIRPα mAbs) but not by non–SIRPα-CD47–interacting antibodies (like anti–Mac-1) or non-Fc–containing SIRPα-CD47 blockers [F(ab)’2 fragment of CC2C6].

Together, our results suggested that if NADPH oxidase activation was engaged through FcR stimulation, then it was simultaneously controlled by the blockade of SIRPα. Considering the potential cytotoxicity of ROS, this tight control is not surprising. Support for this hypothesis was demonstrated by the induction of ROS in PMNs cocultivated with opsonized targets either deficient for CD47 or where engagement of SIRPα by CD47 was blocked by recombinant SIRPα protein. Such control of SIRPα on NADPH oxidase activation was previously shown during myeloid cell differentiation by the restriction of the expression of the gp91^Phox^ subunit of NADPH oxidase (15). The mechanism may be different in mature PMNs considering the short time of response to FcR stimulation and the basal expression of gp91^Phox^ in mature cells and will deserve further studies.

Although our results confirmed the activation of NADPH oxidase in FcR stimulation previously proposed by van Spriel *et al.* (7), recent work based on PMNs from NADPH oxidase–deficient patients claimed that trogoptosis, a mechanism resulting from lytic processes induced by trogocytosis, was solely responsible for ADCC of PMNs (10). Our results do not contradict this statement. The authors reported the use of high ratios of effector to target, suggesting that ADCC without blockade of SIRPα is efficient provided that many PMNs focused on one target. Ratios of 50:1 are used to kill 30% of SKBR3 breast cancer cells opsonized with Trastuzumab (10, 12), and killing increases only two-fold on a CD47-deficient target. On the contrary, trogocytosis at low ratios of PMN to CLL-B cells opsonized with anti-CD20 mAbs does not induce significant death (11) unless SIRPα is blocked allowing activation of NADPH oxidase and strong killing, as we show here for ratios between 3 and 0.5 PMN to 1 target cell.

Hence, cytotoxicity can either result from trogocytosis alone or ROS alone but when the two mechanisms cooperate a single PMN becomes a potent killer. It is tempting to speculate that the spill of ROS into the intracellular milieu of targets through membrane holes created by trogocytosis is the mechanism of optimal function of PMNs. This relationship between the number of PMNs focusing on a target and underlying mechanisms of killing opens new perspectives on the fundamental biology of PMNs where SIRPa-CD47 appears as a key regulator of PMN capacity to differentiate between physiological trogocytosis and cytotoxicity by unleashing trogocytosis and ROS.

The new information brought by our work describing the role of NADPH oxidase activation during ADCC by PMN and its regulation by SIRPα might help to design new agents to enhance myeloid cell function in the treatment of cancer while limiting adverse effects on healthy cells. Although ROS are expected to be released into tumor cells carrying opsonized by tumor-specific therapeutic antibodies triggering trogocytosis, collateral damages resulting from local production of ROS could also suppress T cells infiltrated in the tumor. This could be further exacerbated by the use of non-tumor-specific anti-CD47 antibodies triggering the potent combination of PMN-mediated cytotoxicity described herein. Targeting CD47 with non-FcR activating agents or directly targeting SIRPα could avoid an important part of the killing of non-tumor cells bearing CD47 while preserving the tumor cytotoxic capacity of PMN.

This work was undertaken following the finding that PMN-MDSCs devoid of SIRPα in patients with metastatic melanoma demonstrated a strong capacity to kill T cells using trogocytosis and production of ROS, without *in vitro* activation (26). Activated mature high-density PMNs reproduce such behavior once SIRPα is neutralized. This builds on previous studies pointing to the central role of SIRPα in controlling many aspects of PMNs biology and warrants further studies to elucidate the underlying mechanisms to further refine immunotherapy activity in the immune microenvironment.

## Data Availability Statement

The original contributions presented in the study are included in the article/**Supplementary Material**. Further inquiries can be directed to the corresponding authors.

## Author Contributions

FG-R, TM, JN, and DO designed research; FG-R, VL, and TM performed research; TM, XM, and YC contributed new reagents; FG-R and TM analyzed data; and FG-R, TM, JN, and DO wrote the paper. All authors contributed to the article and approved the submitted version.

## Funding

This work was supported by the National Cancer Institute (1U01CA218259 and R37 CA218259; TM) and the Fondation ARC for Cancer Research (COVID202001312); the team “Immunity and Cancer” was labeled by the Fondation pour la Recherche Médicale “Equipe FRM DEQ20180339209”. VL was supported by a doctoral fellowship from Aix-Marseille Université, then by the Fondation ARC. DO is a Senior Scholar of the Institut Universitaire de France.

## Conflict of Interest

DO is a cofounder of Imcheck Therapeutics, Alderaan Biotechnology, and Emergence Therapeutics. TM is a co-owner of Paradigm Shift Therapeutics.

The remaining authors declare that the research was conducted in the absence of any commercial or financial relationships that could be construed as a potential conflict of interest.

## Publisher’s Note

All claims expressed in this article are solely those of the authors and do not necessarily represent those of their affiliated organizations, or those of the publisher, the editors and the reviewers. Any product that may be evaluated in this article, or claim that may be made by its manufacturer, is not guaranteed or endorsed by the publisher.
